# Literary Items

**Published:** 1889-04

**Authors:** 


### Literary Items.

Messrs. J. B. Lippincott & Company announce to the profession the publication of a Cyclopædia of the Diseases of Children, medical and surgical, by American, British, and Canadian authors, edited by John M. Keating, M.D., in four imperial octavo volumes; to be sold by subscription only. The first volume will be issued early in April, and the subsquent volumes at short intervals.

A thorough knowledge of the diseases of children is a matter of the greatest importance to most physician, and as this is the only work of the kind that has been published in English, it will be invaluable as a text-book and work of reference for the busy practitioner.

